# National cohort and meteorological data based nested case–control study on the association between air pollution exposure and thyroid cancer

**DOI:** 10.1038/s41598-021-00882-7

**Published:** 2021-11-03

**Authors:** Sung Joon Park, Chanyang Min, Dae Myoung Yoo, Hyo Geun Choi

**Affiliations:** 1 Department of Otorhinolaryngology-Head and Neck Surgery, Chung-Ang University College of Medicine, 102 Heukseok-ro, Dongjak-gu, Seoul, 06973 Republic of Korea; 2 Hallym Data Science Laboratory, Hallym University College of Medicine, 22, Gwanpyeong-ro 170beon-gil, Dongan-gu, Anyang-si, Gyeonggi-do 14068 Republic of Korea; 3 Department of Otorhinolaryngology‑Head and Neck Surgery, Hallym University Sacred Heart Hospital, Hallym University College of Medicine, 22, Gwanpyeong-ro 170beon-gil, Dongan-gu, Anyang-si, Gyeonggi-do 14068 Republic of Korea

**Keywords:** Risk factors, Thyroid diseases, Thyroid cancer, Thyroid cancer

## Abstract

The objective of this study was to evaluate the influence of exposure to meteorological conditions, including air pollution, on thyroid cancer. A nested case–control study was conducted utilizing 4632 patients with thyroid cancer and 18,528 control subjects who were matched at a 1:4 ratio by age group, sex, income, and region of residence. Korean National Health Insurance Service-Health Screening Cohort data from 2002 to 2015 were used. Odds ratios (ORs) with 95% confidence intervals (CIs) were calculated for thyroid cancer correlated with meteorological and air pollution exposure over a moving average of 3 years before the index dates. For all participants, the adjusted ORs associated with relative humidity (1.01, 95% CI 1.00–1.03, *P* value = 0.023), ambient atmospheric pressure (1.02, 95% CI 1.01–1.03, *P* value < 0.001), and sunshine duration (1.17, 95% CI 1.04–1.31, *P* value = 0.007) indicated correlations with the occurrence of thyroid cancer; however, these results were inconsistent in the subgroup analyses. Overall, exposure to nitrogen dioxide (NO_2_) (1.33, 95% CI 1.24–1.43, *P* value < 0.001) and particulate matter (PM_10_) (0.64, 95% CI 0.60–0.69, *P* value < 0.001) were related to thyroid cancer. These relationships persisted in the subgroup analyses. In conclusion, thyroid cancer occurrence was positively associated with NO_2_ exposure and negatively associated with PM_10_ exposure.

## Introduction

Thyroid cancer is an endocrine tumor with the highest occurrence, and its incidence has increased in recent decades^1^. Consequently, thyroid cancer is expected to be the fourth most common cancer in the USA by 2030^2^. Increasing trends in thyroid cancer incidence have also been reported in China, Japan, and Korea^3^. The reason for these dramatic increasing trends is unclear. Researchers have postulated that overidentification or overdiagnosis of clinically occult, asymptomatic tumors as well as recent advancements in diagnostic technologies are responsible for the increase^4,5^. However, it is likely that other factors, such as environmental factors, lifestyle, family history and comorbidities, have significant roles in thyroid cancer development^6,7^.

Several environmental factors suggested to be endocrine disruptors have been associated with the occurrence of thyroid cancer. Exposure to certain chemical pollutants, such as decabromodiphenyl ether, which is a congener of polybrominated diphenyl ethers in flame retardants^8^, di(2-ethylhexyl)phthalate, which is a metabolite of phthalate in plastic materials and a plasticizer^9^, organochlorine pesticides^10^, and nitrates^11^ have been reported to be associated with thyroid cancer. In addition, exposures to certain environmental conditions have also been reported to have carcinogenic effects on the thyroid gland. For example, physical factors, such as ionizing radiation exposure^11^; geographic factors, such as living in a volcanic environment^12^; and a cold climate^14^ have been reported to be risk factors for thyroid cancer.

However, only a few reports support the potential association between air pollution and thyroid cancer, and no previous articles have been reported from Eastern Asian countries, such as Korea or Japan. When we searched PubMed using the terms ‘air pollution’ and ‘thyroid cancer’, a total of 10 relevant articles written in English were found between January 1991 and September 2021. Of these 10 articles, only two articles specifically evaluated the association between air pollution and thyroid cancer. An ecological study performed within European Union member states revealed a significant association between thyroid cancer incidence in men and benzo(k)fluoranthene (r2 = 0.2142, *P* value = 0.042) or hexachlorocyclohexane (r2 = 0.9993, *P* value = 0.0166) exposure^13^, and a more recently reported case–control study performed in the United States of America revealed that a 5 μg/m^3^ increase in exposure to fine particulate matter (diameter ≤ 2.5 μm, PM_2.5_) concentrations over 2 years (adjusted odds ratio [OR] = 1.18, 95% confidence interval [CI] 1.00–1.40) and 3 years (adjusted OR = 1.23, 95% CI 1.05–1.44) was associated with increased odds of papillary thyroid cancer^15^. A further literature review revealed only two more articles reporting the direct correlation between air pollution and thyroid cancer. Interestingly, air pollution originating from industrial waste gas was significantly positively associated with thyroid cancer occurrences in a Chinese population^16^, and overall exposure to urban PM_10_ showed a significant correlation with thyroid cancer incidence in Brazil^17^.

Because of the potential existence of regional variations in air pollution and racial differences in the response to air pollutants, we sought to perform an additional investigation of the association between air pollution and thyroid cancer in a Korean population by using a national cohort database. It was hypothesized in the present study that meteorological circumstances and air pollution may affect the incidence of thyroid cancer. A nested case–control study was performed to verify our hypothetical correlation between exposure to various meteorological parameters and air pollution for 3 years prior to the date of the initial diagnosis of thyroid cancer and the occurrence of thyroid cancer.

## Results

### General characteristics of the participants

The male to female ratio among the study participants was 0.27 (4965–18,195). Because the age groups were categorized using 5-year intervals, the mean age could not be defined (Table 1). The age group-, sex-, income level-, and region of residence-matched control group showed significant differences in the rate of obesity, smoking status, alcohol consumption habit, CCI scores, total cholesterol levels, SBP, and DBP compared to the thyroid cancer group. These factors were adjusted for in the subsequent analyses. Meteorological and air pollution parameters for 3 years prior to the index date showed significant differences in mean temperature, highest temperature, lowest temperature, ambient atmospheric pressure, sunshine duration, rainfall, O_3_, CO, and PM_10_ between the thyroid cancer and control groups (Table 1).

**Table 1:** General characteristics of participants.

Characteristics	Total participants		*P* value
	Thyroid cancer	Control	
Age group (years old, n, %)		1.000	
45–49	520 (11.2)	2080 (11.2)	
50–54	1335 (28.8)	5340 (28.8)	
55–59	1142 (24.7)	4568 (24.7)	
60–64	776 (16.8)	3104 (16.8)	
65–69	475 (10.3)	1900 (10.3)	
70–74	277 (6.0)	1108 (6.0)	
75–79	83 (1.8)	332 (1.8)	
80–84	23 (0.5)	92 (0.5)	
≥ 85	1 (0.0)	4 (0.0)	
Sex (n, %)		1.000	
Male	993 (21.4)	3972 (21.4)	
Female	3639 (78.6)	14,556 (78.6)	
Income (n, %)		1.000	
1 (lowest)	604 (13.0)	2416 (13.0)	
2	515 (11.1)	2060 (11.1)	
3	733 (15.8)	2932 (15.8)	
4	959 (20.7)	3836 (20.7)	
5 (highest)	1821 (39.3)	7284 (39.3)	
Region of residence (n, %)		1.000	
Urban	2216 (47.8)	8864 (47.8)	
Rural	2416 (52.2)	9664 (52.2)	
Obesity (BMI, kg/m^2^, n, %)		< 0.001*	
< 18.5 (underweight)	64 (1.4)	393 (2.1)	
≥ 18.5 to < 23 (normal)	1524 (32.9)	6911 (37.3)	
≥ 23 to < 25 (overweight)	1315 (28.4)	5016 (27.1)	
≥ 25 to < 30 (obese I)	1533 (33.1)	5613 (30.3)	
≥ 30 (obese II)	196 (4.2)	595 (3.2)	
Smoking status (n, %)		< 0.001*	
Nonsmoker	4030 (87.0)	15,785 (85.2)	
Past smoker	348 (7.5)	1145 (6.2)	
Current smoker	254 (5.5)	1598 (8.6)	
Alcohol consumption (n, %)		0.008*	
< 1 time a week	3515 (75.9)	14,398 (77.7)	
≥ 1 time a week	1117 (24.1)	4130 (22.3)	
Charlson comorbidity index (n, %)		< 0.001*	
0	2882 (62.2)	14,597 (78.8)	
1	755 (16.3)	2155 (11.6)	
2	279 (6.0)	903 (4.9)	
3	105 (2.3)	397 (2.1)	
≥ 4	611 (13.2)	476 (2.6)	
Total cholesterol (mg/dL, mean, SD)	199.4 (37.8)	202.1 (37.6)	< 0.001*
SBP (mmHg, mean, SD)	124.1 (15.5)	123.3 (15.6)	0.003*
DBP (mmHg, mean, SD)	77.2 (10.2)	76.6 (10.2)	0.001*
Fasting blood glucose (mg/dL, mean, SD)	98.5 (24.8)	0.061	
Meteorological and air pollution data (mean, SD)			
Mean temperature for 3 years (1095 days) (°C)	12.9 (1.2)	12.9 (1.2)	0.005*
Highest temperature for 3 years (1095 days) (°C)	18.0 (1.1)	18.0 (1.1)	0.008*
Lowest temperature for 3 years (1095 days) (°C)	8.6 (1.6)	8.5 (1.7)	0.022*
Temperature range for 3 years (1095 days) (°C)	9.4 (1.4)	9.4 (1.4)	0.556
Relative humidity for 3 years (1095 days) (%)	65.5 (4.6)	65.5 (4.4)	0.424
Ambient atmospheric pressure for 3 years (1095 days) (hPa)	1006.4 (4.0)	1006.1 (4.4)	< 0.001*
Sunshine duration for 3 years (1095 days) (hr)	5.8 (0.4)	5.8 (0.3)	0.003*
Rainfall for 3 years (1095 days) (mm)	8.7 (0.9)	8.6 (0.9)	0.019*
SO_2_ for 3 years (1095 days) (ppb)	5.3 (1.0)	5.3 (1.0)	0.073
NO_2_ for 3 years (1095 days) (ppb)	24.1 (8.7)	24.2 (8.7)	0.495
O_3_ for 3 years (1095 days) (ppb)	23.7 (4.6)	23.4 (4.5)	0.001*
CO for 3 years (1095 days) (ppb)	525.4 (80.0)	530.7 (80.6)	< 0.001*
PM_10_ for 3 years (1095 days) (μg/m^3^)	51.9 (6.4)	52.7 (6.5)	< 0.001*

*BMI* body mass index (kg/m^2^); *ppb* parts per billion; *SD* standard deviation.

*Chi-square test or independent *t* test. Significance at *P* < 0.05.

#### Meteorological conditions, air pollution parameters, and thyroid cancer

All of the meteorological and air pollution parameters except temperature range, mean relative humidity, mean SO_2_ and mean NO_2_ concentration were significantly associated with thyroid cancer according to the crude ORs (Table 2). In model 1, the mean temperature (1.04, 95% CI 1.01–1.07), lowest temperature (1.03, 95% CI 1.00–1.06, *P* value = 0.026), ambient atmospheric pressure (1.02, 95% CI 1.01–1.03), sunshine duration (1.21, 95% CI 1.09–1.34), rainfall (1.09, 95% CI 1.04–1.14), SO_2_ (0.69, 95% CI 0.48–0.98), O_3_ (1.17, 95% CI 1.07–1.28), CO (0.42, 95% CI 0.27–0.64), and PM_10_ (0.81, 95% CI 0.77–0.85) were significantly associated with the occurrence of thyroid cancer (Table 2).

**Table 2:** Crude and adjusted odds ratios (95% confidence interval, CI) of meteorological conditions and air pollution parameters (mean of 3 years [1095 days] before index date) for thyroid cancer.

Characteristics		Odds ratio for thyroid cancer (95% CI)					
		Crude^†^	*P* value	Model 1^†,‡^	*P* value	Model 2^†,§^	*P* value
Mean temperature (°C)		1.05 (1.02–1.08)	0.002*	1.04 (1.01–1.07)	0.020*		
Highest temperature (°C)		1.04 (1.01–1.07)	0.008*	1.03 (1.00–1.06)	0.059		
Lowest temperature (°C)		1.04 (1.01–1.07)	0.003*	1.03 (1.00–1.06)	0.026*		
Temperature range (°C)		0.98 (0.95–1.02)	0.355	0.99 (0.95–1.02)	0.430		
Relative humidity (%)		1.01 (1.00–1.02)	0.240	1.00 (0.99–1.01)	0.676	1.01 (1.00–1.03)	0.023*
Ambient atmospheric pressure (hPa)		1.02 (1.01–1.03)	< 0.001*	1.02 (1.01–1.03)	< 0.001*	1.02 (1.01–1.03)	< 0.001*
Sunshine duration (hr)		1.18 (1.07–1.30)	0.001*	1.21 (1.09–1.34)	< 0.001*	1.17 (1.04–1.31)	0.007*
Rainfall (mm)		1.07 (1.02–1.12)	0.003*	1.09 (1.04–1.14)	< 0.001*		
SO_2_ (0.01 ppm)		0.71 (0.50–1.00)	0.052	0.69 (0.48–0.98)	0.040*		
NO_2_ (0.01 ppm)		0.98 (0.94–1.03)	0.388	1.00 (0.96–1.05)	0.924	1.33 (1.24–1.43)	< 0.001*
O_3_ (0.01 ppm)		1.19 (1.10–1.30)	< 0.001*	1.17 (1.07–1.28)	< 0.001*		
CO (ppm)		0.42 (0.28–0.63)	< 0.001*	0.42 (0.27–0.64)	< 0.001*		
PM_10_ (10 μg/m^3^)		0.81 (0.77–0.85)	< 0.001*	0.81 (0.77–0.85)	< 0.001*	0.64 (0.60–0.69)	< 0.001*

*CCI* Charlson comorbidity index; *DBP* diastolic blood pressure; *SBP* systolic blood pressure.

* Conditional logistic regression model, significance at *P* < 0.05.

^†^ Stratified model for age group, sex, income, and region of residence.

^‡^Model 1 was adjusted for total cholesterol, SBP, DBP, fasting blood glucose, obesity, smoking status, alcohol consumption, and CCI score.

^§^Model 2 was adjusted for total cholesterol, SBP, DBP, fasting blood glucose, obesity, smoking status, alcohol consumption, CCI score, temperature range, relative humidity, ambient atmospheric pressure, sunshine duration, SO_2_, NO_2_, O_3_, CO, and PM_10_ using the forward selection method.

In model 2, relative humidity (1.01, 95% CI 1.00–1.03, *P* value = 0.023), ambient atmospheric pressure (1.02, 95% CI 1.01–1.03), sunshine duration (1.17, 95% CI 1.04–1.31), NO_2_ (1.33, 95% CI 1.24–1.43), and PM_10_ (0.64, 95% CI 0.60–0.69) were significantly associated with the occurrence of thyroid cancer (Table 2).

#### Subgroup analysis according to age, sex, income, and region of residence

The model 2 outcomes of the subgroup analyses are summarized in Table 3. In the subgroup analysis by age, various meteorological factors and air pollutants were significantly associated with thyroid cancer. NO_2_ showed a higher OR in the young age group (age < 60 years old: 1.52, 95% CI 1.33–1.73) than in the old age group (age ≥ 60 years old: 1.03, 95% CI 1.02–1.04), and PM_10_ also showed a higher OR in the young age group (age < 60 years old: 0.66, 95% CI 0.59–0.73) than in the old age group (age ≥ 60 years old: 0.60, 95% CI 0.54–0.67). However, the positive association of NO_2_ and negative association of PM_10_ were consistent in both age groups. Inconsistent associations of meteorological factors and other air pollutants with thyroid cancer were observed in the subgroup analyses by sex, income, and region of residence. NO_2_ showed a higher OR in males (1.77, 95% CI 1.52–2.05) than in females (1.22, 95% CI 1.13–1.31), in the high-income group (1.36, 95% CI 1.25–1.47) than in the low-income group (1.18, 95% CI 1.06–1.31), and in the rural population (2.08, 95% CI 1.76–2.46) than in the urban population (1.27, 95% CI 1.08–1.50). However, NO_2_ showed a consistent positive association with thyroid cancer regardless of sex, income, or region of residence. In addition, PM_10_ showed a higher OR in females (0.69, 95% CI 0.63–0.75) than in males (0.51, 95% CI 0.43–0.60), in the low-income group (0.66, 95% CI 0.59–0.75) than in the high-income group (0.64, 95% CI 0.58–0.70), and in the rural population (0.70, 95% CI 0.57–0.86) than in the urban population (0.53, 95% CI 0.46–0.61). However, PM_10_ showed a consistent negative association with thyroid cancer regardless of sex, income, or region of residence.

**Table 3:** Subgroup analyses of crude and adjusted odd ratios (95% confidence interval, CI) of meteorological conditions and air pollution parameters (mean of 3 years [1095 days] before index date) for thyroid cancer according to age, sex, income, and region of residence.

Characteristics	Odds ratio for thyroid cancer (95% CI)	
	Model 2 †‡	*P* value
Age < 60 years old (n = 14,985)		
Temperature range (°C)	1.12 (1.06–1.19)	< 0.001*
Ambient atmospheric pressure (hPa)	1.03 (1.02–1.04)	< 0.001*
NO_2_ (0.01 ppm)	1.52 (1.33–1.73)	< 0.001*
O_3_ (0.01 ppm)	1.46 (1.10–1.94)	0.010*
PM_10_ (10 µg/m^3^)	0.66 (0.59–0.73)	< 0.001*
Age ≥ 60 years old (n = 8175)		
Ambient atmospheric pressure (hPa)	1.07 (1.01–1.12)	0.012*
NO_2_ (0.01 ppm)	1.03 (1.02–1.04)	< 0.001*
O_3_ (0.01 ppm)	1.40 (1.28–1.54)	< 0.001*
PM_10_ (10 µg/m^3^)	0.60 (0.54–0.67)	< 0.001*
Males (n = 4965)		
Relative humidity (%)	1.05 (1.03–1.08)	< 0.001*
NO_2_ (0.01 ppm)	1.77 (1.52–2.05)	< 0.001*
PM_10_ (10 μg/m^3^)	0.51 (0.43–0.60)	< 0.001*
Females (n = 18,195)		
Ambient atmospheric pressure (hPa)	1.03 (1.02–1.04)	< 0.001*
Sunshine duration (hr)	1.15 (1.02–1.30)	0.022*
NO_2_ (0.01 ppm)	1.22 (1.13–1.31)	< 0.001*
PM_10_ (10 μg/m^3^)	0.69 (0.63–0.75)	< 0.001*
Low income (n = 9260)		
Ambient atmospheric pressure (hPa)	1.03 (1.02–1.04)	< 0.001*
NO_2_ (0.01 ppm)	1.18 (1.06–1.31)	0.003*
PM_10_ (10 μg/m^3^)	0.66 (0.59–0.75)	< 0.001*
High income (n = 13,900)		
Ambient atmospheric pressure (hPa)	1.02 (1.01–1.04)	< 0.001*
NO_2_ (0.01 ppm)	1.36 (1.25–1.47)	< 0.001*
PM_10_ (10 μg/m^3^)	0.64 (0.58–0.70)	< 0.001*
Urban (n = 11,080)		
Relative humidity (%)	0.97 (0.96–0.99)	< 0.001*
NO_2_ (0.01 ppm)	1.27 (1.08–1.50)	0.004*
O_3_ (0.01 ppm)	2.57 (1.86–3.55)	< 0.001*
CO (ppm)	45.03 (14.26–142.22)	< 0.001*
PM_10_ (10 μg/m^3^)	0.53 (0.46–0.61)	< 0.001*
Rural (n = 12,080)		
Temperature range (°C)	1.19 (1.08–1.30)	< 0.001*
Relative humidity (%)	1.08 (1.05–1.10)	< 0.001*
NO_2_ (0.01 ppm)	2.08 (1.76–2.46)	0.016*
O_3_ (0.01 ppm)	1.58 (1.09–2.29)	< 0.001*
CO (ppm)	0.05 (0.02–0.13)	0.001*
PM_10_ (10 μg/m^3^)	0.70 (0.57–0.86)	0.007*

*CCI* Charlson comorbidity index; *DBP* diastolic blood pressure; SBP, systolic blood pressure.

*Conditional logistic regression model, significance at *P* < 0.05.

^†^Stratified model for age group, sex, income, and region of residence.

^‡^Model 2 was adjusted for total cholesterol, SBP, DBP, fasting blood glucose, obesity, smoking status, alcohol consumption, CCI score, temperature range, relative humidity, ambient atmospheric pressure, sunshine duration, SO_2_, NO_2_, O_3_, CO, and PM_10_ using the forward selection method.

## Discussion

In the present study, the adjusted ORs associated with relative humidity (1.01, 95% CI 1.00–1.03), ambient atmospheric pressure (1.02, 95% CI 1.01–1.03), and sunshine duration (1.17, 95% CI 1.04–1.31) indicated positive correlations with the occurrence of thyroid cancer. However, these results were inconsistent in the subgroup analyses. Overall, NO_2_ (1.33, 95% CI 1.24–1.43) and PM_10_ (0.64, 95% CI 0.60–0.69) were significantly associated with thyroid cancer, and these relationships were consistently significant in the subgroup analyses. Only a few studies have investigated the relationship between air pollution and thyroid cancer. Our study is the first to comprehensively evaluate the association between air pollution and thyroid cancer with adjustments for various meteorological conditions using a nested case–control study design.

Because NO_2_ exposure induces oxidative stress and systemic inflammation and is involved in other pathogenic mechanisms, it has been recognized as a carcinogen^11^. Giannoula et al. showed in an ecological study that certain components of air pollutants may increase thyroid cancer incidence^13^. NO_2_ is a reactive compound in polluted air that has carcinogenic potential. It can initiate free radical reactions by interacting with unsaturated fatty acids and inducing the autooxidation of organic compounds^18,19^. A significant association between chronic exposure to NO_2_ and lung cancer (OR 1.30, 95% CI 1.02–1.66) has been reported^20^, and genomic instability and thyroid hypertrophy through the overproduction of cellular reactive oxygen species (ROS) have been postulated as potential mechanisms by which NO_2_ induces thyroid cancer^11^. Therefore, the positive association between NO_2_ and the occurrence of thyroid cancer shown in our results may also be related to NO_2_-induced oxidative stress and the overproduction of cellular ROS.

In addition, NO_2_ is known as a potential endocrine-disrupting chemical. In a previous report, an increased NO_2_ concentration in air pollutants showed a strong correlation with elevated odds of primary hypothyroidism (Spearman correlation coefficients; adolescent female = 0.94, adolescent male = 0.94)^21^. In addition, annual average exposure to ambient NO_2_ was significantly correlated with a decrease in the free thyroxine (FT4) concentration (β-coefficients [SE]: − 0.0072 [0.0024], *P* value = 0.003) and an increase in thyroid-stimulating hormone (TSH) (β-coefficients [SE]: 0.0131 [0.0053], *P* value = 0.01)^22^. In animals, the release of excess TSH has been reported to induce various thyroid diseases, including thyroid cancer^23^. Moreover, there is evidence of TSH receptor signaling as an oncogenic pathway in developing thyroid cancer^23^. Based on these findings, we have also postulated that consistent exposure to NO_2_ in air pollutants can increase circulating TSH, resulting in increased TSH receptor signaling and an increased incidence of thyroid cancer.

Although higher concentrations of PM_2.5_ and PM_10_ (surrogate indicators for air pollution) have been reported to be disruptive to the endocrine system and carcinogenic in humans^15,24^, previous studies showed inconsistent results of the association between PM and thyroid function. Cong reported that exposure to waste gas-emitted ambient air pollution was positively correlated with an increased thyroid cancer incidence (r_s_ = 0.716, *P* value < 0.001)^16^. However, it was reported that outdoor PM_2.5_ exposure was not correlated with death due to most non-lung cancers, such as thyroid cancer (hazard ratio 0.62, 95% CI 0.34–1.12)^25^. Additionally, the PM_10_ concentration was not strongly associated with an elevated risk of primary hypothyroidism (Spearman correlation coefficients; adult female: 0.89, adult male: 0.89)^21^, and the average level of PM_10_ exposure was positively associated with the TSH level in only men (β-coefficient [SE]: 0.0159 [0.0074], *P* value = 0.03); additionally, no significant association with the overall change in the FT4 (β-coefficient [SE]: − 0.0029 [0.0030], *P* value = 0.33) or TSH level (β-coefficient [SE]: 0.0103 [0.0066], *P* value = 0.12) was observed^22^. Ghassabian et al. reported that there were no significant associations between PM_10_ and decreased FT4 in pregnant women (OR 1.18, 95% CI 0.93–1.48), and only high exposure to PM_2.5_ was related to hypothyroxinemia (OR 1.21, 95% CI 1.00–1.47)^26^.

Considering the previously reported carcinogenic effects of PM, our study results unexpectedly showed a negative association between PM_10_ and thyroid cancer. Yanagi et al. reported that the statistical correlation between overall exposure to urban PM_10_ and thyroid cancer incidence was high and significant^17^. The induced systemic inflammation and the immune response to autoantigens resulting in the production of ROS have been proposed as mechanisms of PM carcinogenesis in thyroid cancer patients^11^. However, our study results support the following study results regarding the endocrine-disrupting effect of PM_10_. Oziol et al. reported that ambient air in French urban areas had thyroid receptor alpha-1 agonistic effects without competitive effects with regard to T3-dependent transcriptional activity^27^. Similarly, Nováková et al. conducted an in vitro experiment and found that exposure to PM_10_ in ambient air significantly increased thyroid receptor-mediated activity, and exposure to a submicrometer fraction of PM_10_, namely, particles sized 0.49–0.95 µm, was associated with the highest activity^28^. Additionally, Dong et al. reported that PM_2.5_ exposure was significantly connected with decreased serum levels of triiodothyronine (T3), T4, and TSH (*P* value < 0.05) in an in vivo female rat model^29^. The authors also reported that PM_2.5_ exposure repressed the biosynthesis and biotransformation of thyroid hormones (THs) by activating the hypothalamic-pituitary-thyroid (HPT) axis and inducing hepatic transthyretin^29^. Moreover, Zeng et al. conducted a retrospective cross-sectional study and concluded that a 10 µg/m^3^ increase in PM_2.5_ was associated with a 0.12 pmol/L decrease in FT4 and a 0.07 pmol/L increase in FT3, and the FT4/FT3 ratio was inversely associated with PM_2.5_ (coefficient: − 0.06, *P* value < 0.01)^30^. Based on these findings, we postulate that PM_10_ decreases the incidence of thyroid cancer through increased thyroid receptor-mediated activity by increasing FT3, which results in decreased TSH levels, initiation of the HPT axis, and induction of hepatic transthyretin.

Several limitations are present regarding the interpretation of the present results. First, although age group, sex, income, and region of residence were matched and adjusted for, lifestyle factors, such as obesity (BMI), tobacco smoking, and alcohol consumption habits, as well as the CCI score, total cholesterol level, SBP, and DBP, were not matched between the two groups in this study. Moreover, family history of thyroid cancer, dietary habits, physical activity, and history of radiation exposure were not surveyed in this study. Second, interactions of variable combinations of air pollutants and meteorological conditions could not be excluded. Although multiple air pollutants, namely, SO_2_, NO_2_, O_3_, CO, and PM_10,_ were considered in this study, the effect of PM_2.5_ was not analyzed because PM_2.5_ was not measured before 2015 in Korea. Third, potential errors of meteorological or air pollutant exposure category was inevitable in this study. Since the estimation of meteorological and air pollutant exposure was conducted by the region of residence of each participant rather than by taking individual patterns of activity and living sphere into account, intersubject variability was possible. Fourth, the positive associations of relative humidity, ambient atmospheric pressure, and sunshine duration were not retained or consistent in the subgroup analyses. There is insufficient evidence to explain the effects of sex, age, socioeconomic status, and site of residence on the association of meteorological conditions and thyroid cancer. Thus, further studies to evaluate associations between various meteorological conditions and exposure to air pollutants in the abovementioned subgroups are required to investigate the exact effect of each component on thyroid cancer. Fifth, information about indoor air pollutant exposure was unavailable. For example, exposure to indoor NO_2_ from fuel-fired apparatuses and stoves and cigarette smoking might have influenced the present results. Sixth, 3 years prior to diagnosis may not be sufficient for the development of thyroid cancer. However, we tried to overcome this limitation by including the region of residence, which reflected continuous exposure to certain air pollutants and meteorological conditions that affected the occurrence of thyroid cancer, as a cofactor. Seventh, as this study used NHIS data, we could not include thyroid cancer patients who did not visit any medical clinic or hospital. Additionally, those with undetected thyroid cancer might have been included among the control participants. Finally, because meteorological conditions and air pollution levels are distinct by region, the comprehension of this study outcomes may be limited to the Korean Peninsula. Additional studies in distinct geographical regions are needed to contemplate specific characteristics in variable regions.

In conclusion, the mean concentration of NO_2_ in the 3 years before the onset of thyroid cancer was significantly related to an increased risk of thyroid cancer in the thyroid cancer group, while the mean concentration of PM_10_ was associated with a significantly decreased risk in the thyroid cancer group compared to that in the control group. In addition, the positive association of NO_2_ and the negative association of PM_10_ was consistent regardless of age group, sex, income level, or region of residence. Therefore, consistent efforts to decrease NO_2_ in the ambient air should be made to relieve the worldwide increase in the incidence of thyroid cancer, while further multinational ecological studies should be performed to ascertain the precise relationship between PM_10_ and thyroid cancer.

## Methods

### Ethics

The ethics committee of Hallym University (2019–10-022) permitted this study. Written informed consent was waived by the Institutional Review Board of Hallym University. This study was performed in accordance with the Declaration of Helsinki and followed the STROBE (Strengthening the Reporting of Observational Studies in Epidemiology) guidelines for reporting.

#### Study population and participant selection

We analyzed Korean National Health Insurance Service-Health Screening Cohort (NHIS-HEALS) data (which is not publicly available data and can be accessed only after acquiring permission from IRB, passing the internal screening held by the National Health Insurance Service of Korea, and paying the cost to acquire the data) between 2002 and 2015 and meteorological and air pollution parameters from the meteorological administration, as described in the supplementary material (S1 description) and in previous studies^31,32^.

**Figure 1:**
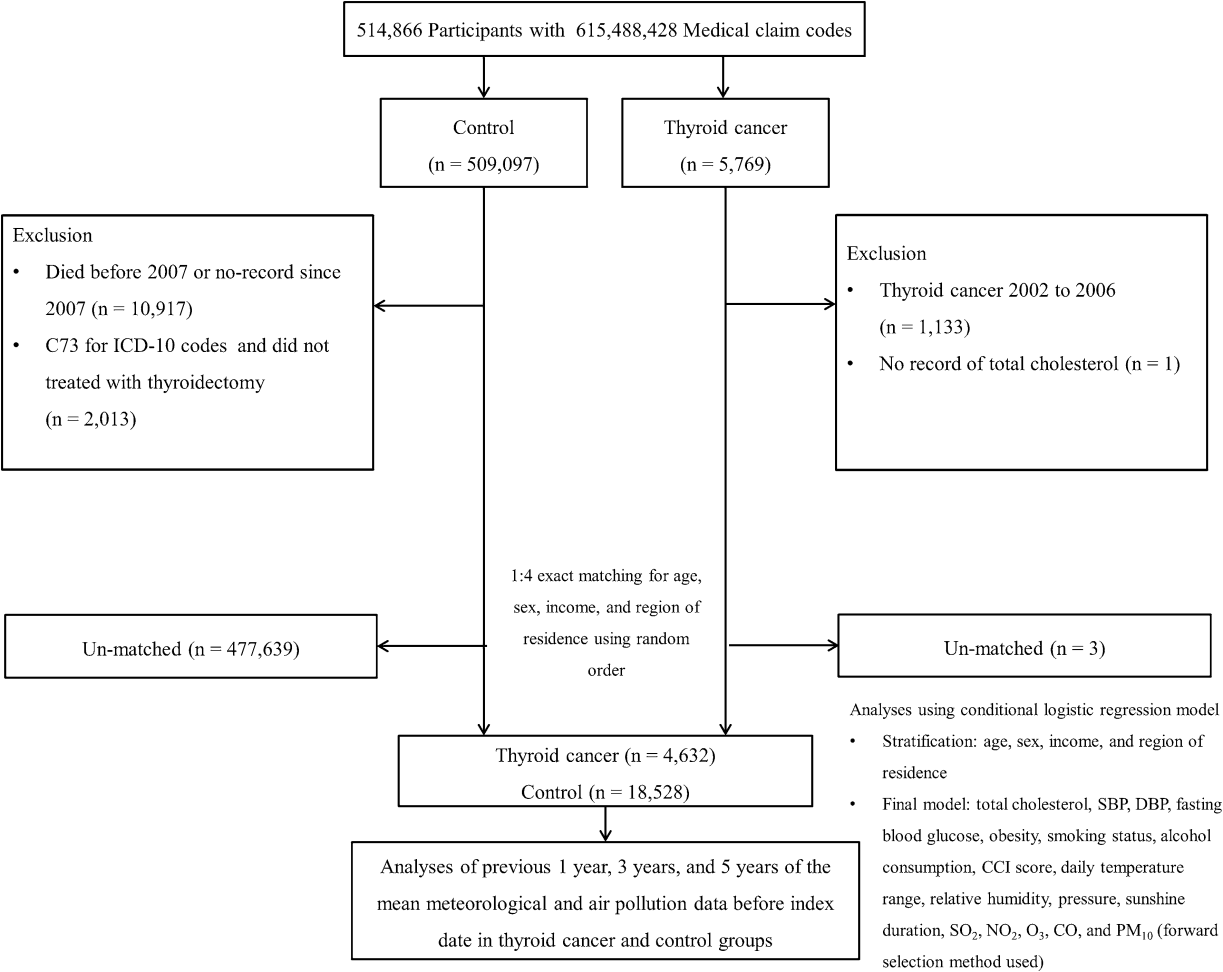
A schematic illustration of the participant selection process that was used in the present study. Of a total of 514,866 participants, 4632 thyroid cancer participants were matched with 18,528 control participants for age, sex, income, and region of residence. Then, the thyroid cancer and control groups were linked with meteorological data and air pollution data before the index date.

Participants who were diagnosed with thyroid cancer were selected from 514,866 patients with 615,488,428 medical claim codes (n = 5769). To ensure the availability of air pollution exposure data for 5 years before the index date, we excluded thyroid cancer participants who were diagnosed from 2002 to 2006 (n = 1133). The control group comprised participants who did not have thyroid cancer from 2002 to 2015, and they were selected from the total population (n = 509,097). Participants who died before 2007 or had no records since 2007 (n = 10,917) were excluded from the control group. In addition, control participants were excluded if they were diagnosed with the C73 (malignant neoplasm of the thyroid gland) International Classification of Diseases 10^th^ revision (ICD-10) code and did not undergo thyroidectomy (n = 2013). The thyroid cancer group was matched with the control group in a 1:4 ratio by age group, sex, income, and region of residence. The random number method was utilized in selecting the control groups to countervail the selection bias. The time of thyroid cancer diagnosis was referred to as the index date for thyroid cancer patients. A random day within the 1-year period prior to the index date of each thyroid cancer participant was defined as the index date for participants in the control group. Therefore, participants with an index date between 2007 and 2015 were included in the control group. Throughout the matching procedure, 477,639 participants in the control group and 3 participants in the thyroid group were excluded. Consequently, 4632 thyroid cancer participants were matched in a 1:4 ratio with 18,528 control participants (Fig. 1).

The meteorological and air pollution parameters over a moving average of 1 year (365 days), 3 years (1095 days), and 5 years (1825 days) before the index date were investigated and compared among the thyroid cancer group and the control group.

#### Variables

##### Independent variables

Daily mean temperature (°C), highest temperature (°C), lowest temperature (°C), and temperature range (°C), along with relative humidity (%), ambient atmospheric pressure (hPa), sunshine duration (hr), precipitation (mm), SO_2_ (ppm), NO_2_ (ppm), O_3_ (ppm), CO (ppm), and PM_10_ (µg/m^3^) for moving averages of 1 year (365 days), 3 years (1095 days), and 5 years (1825 days) before the index dates, were defined as the independent variables, as described in previous studies^31,32^.

##### Covariates

Patients were stratified into age groups with 5-year intervals: 45–49, 50–54…, and 85 + years old (9 age groups). Income level was stratified into 5 classes as follows: class 1 (lowest income) to class 5 (highest income). The region of residence was stratified into urban (Seoul, Busan, Daegu, Incheon, Gwangju, Daejon, and Ulsan) and rural (Gyeonggi, Gangwon, Chungcheongbuk, Chungcheongnam, Jeollabut, Jeollanam, Gyeongsangbuk, Gyeongsangnam, and Jeju) areas according to the classification of administrative districts by the Korean government, which have been described in a previous study^33^. Tobacco smoking was categorized according to the participant’s current smoking status (nonsmoker, past smoker, or current smoker), alcohol consumption was classified on the basis of the frequency of alcohol consumption (< 1 time a week and ≥ 1 time a week), and obesity regarded by body mass index (BMI, kg/m^2^) was categorized as underweight (< 18.5), normal (≥ 18.5 to < 23), overweight (≥ 23 to < 25), obese I (≥ 25 to < 30), and obese II (≥ 30) as described in previous studies^34,35^. Systolic blood pressure (SBP), diastolic blood pressure (DBP), fasting blood glucose, and total cholesterol levels were measured. The Charlson Comorbidity Index (CCI) was used to assess the underlying comorbidity status^36^, which was rated from 0 (no comorbidities) to 29 (multiple comorbidities), and thyroid cancer was omitted from the rating score.

##### Dependent variable

The diagnosis of thyroid cancer was referred to as participants with ICD-10 code C73 (malignant neoplasm of the thyroid gland) and treatment claim codes for thyroidectomy (P4551, P4552, P4553, P4554, and P4561)^6,7^.

### Statistical analyses

The comparison of general characteristics of two groups was conducted using the chi-square test (independent variables) and the independent t-test (continuous variables). The mean meteorological and air pollution parameters for 3 years (1095 days) before the index date in the two groups were compared using the independent t-test.

To assess the relationship between meteorological and air pollution exposure and thyroid cancer, each parameter was compared between the thyroid cancer group and the control group. The odds ratios (ORs) with 95% confidence intervals (CIs) of each parameter in the thyroid cancer group were evaluated using conditional logistic regression analysis within three different models constructed as follows: a crude (simple) model, model 1 (adjusted for total cholesterol, SBP, DBP, fasting blood glucose, obesity, smoking status, alcohol consumption habit, and CCI score), and model 2 (adjusted for model 1 plus daily temperature range, relative humidity, pressure, sunshine duration, SO_2_, NO_2_, O_3_, CO, and PM_10_ applying the forward selection method). Age group, sex, income, and region of residence were stratified in these analyses. Furthermore, the correlation between each of meteorological conditions and air pollution parameters were statistically analyzed to preclude the effect of collinearity between each covariates in model 2 (Table S2). Among 365 days, 1095 days, and 1825 days of exposure durations, we selected 1095 days as the main exposure term. The results of the other durations are presented in the supplemental files (Tables S3 and S4).

For subgroup analysis, we stratified participants by age (< 60 years old and ≥ 60 years old), sex (males and females), income (low and high income), and region of residence (urban and rural area), and ORs in model 2 were evaluated. The outcomes of the subgroup analyses of other durations of exposure are presented in the supplemental files (Tables S5 and S6).

Two-tailed analyses were performed, and a *P* value less than 0.05 was considered the significance level. SAS version 9.4 (SAS Institute Inc., Cary, NC, USA) was utilized in the statistical analysis.

## Supplementary Information

Supplementary Information.
