# Supplementary material for: National cohort and meteorological data based nested case–control study on the association between air pollution exposure and thyroid cancer
Source: Sci Rep. 2021 Nov 3;11:21562. doi: 10.1038/s41598-021-00882-7 (PMC8566463; doi:10.1038/s41598-021-00882-7)
Supplement: Supplementary file 1 — Supplementary Information. [file 41598_2021_882_MOESM1_ESM.pdf]

National cohort and meteorological data based nested case-control study on the association between air pollution exposure and thyroid cancer

Sung Joon Park,[1] Chanyang Min,[2] Dae Myoung Yoo,[2] Hyo Geun Choi[2,3*]

[1]Department of Otorhinolaryngology-Head and Neck Surgery, Seoul National University Hospital, Seoul National University College of Medicine, Seoul, Republic of Korea.

[2]Hallym Data Science Laboratory, Hallym University College of Medicine, Anyang, Republic of Korea

[3]Department of Otorhinolaryngology-Head & Neck Surgery, Hallym University College of Medicine, Anyang, Republic of Korea.

S1 description: Study Population and Data Collection

This national cohort study relied on data from the Korean National Health Insurance Service-Health Screening Cohort (NHIS-HEALS) (1). The Korean National Health Insurance Service (NHIS) randomly selects approximately 10% of individuals who underwent health examinations from 2002 to 2003 (n = ~515,000) directly from the entire population (n = ~5,150,000). Age and sex specific distributions of the cohort population are described online (2). The details of the methods used to perform these procedures are provided by the National Health Insurance Sharing Service (3).

All insured Koreans who are at least 40 years old and their dependents undergo no-cost biannual health examinations. Each examinee must complete a standard questionnaire in for this health screening program (4). Because all Korean citizens are recognized by a 13-digit resident registration number from birth to death, exact population statistics can be determined using this database. It is mandatory for all Koreans to enroll in the NHIS. All Korean hospitals and clinics use the 13-digit resident registration number to register individual patients in the medical insurance system. Therefore, the risk of overlapping medical records is minimal, even if a patient moves from one place to another. Moreover, all medical treatments in Korea can be tracked without exception using the Korean Health Insurance Review & Assessment (HIRA) system. In Korea, providing a notice of death to an administrative entity is legally required before a funeral can be held, and the cause and date of death are recorded by medical doctors on a death certificate.

This cohort database includes (i) personal information, (ii) health insurance claim codes (procedures and prescriptions), (iii) diagnostic codes using the International Classification of Disease-10 (ICD-10), (iv) death records from the Korean National Statistical Office (using the Korean Standard Classification of disease), (v) socioeconomic data (residence and income), (vi) medical examination data (vii) health examination data (body mass index, BMI), drinking and smoking habits, blood pressure, urinalysis, hemoglobin, fasting glucose, lipid parameters, creatinine, and liver enzymes) for each participant over the period from 2002 to 2015 (3,4).

Meteorological Data

Temperature (˚C), Relative humidity (%), were Spot atmospheric pressure (hPa) data were obtained from the meteorological administration. It was measured by automated synoptic

observing system (ASOS) and manually in 94 places hourly. Quality was controlled following quality inspection manual (5).

SO_2 (ppm), NO_2 (ppm), O_3 (ppm), CO (ppm), and PM_{10} (μg/m^3) data were obtained by the ministry of environment. It was measured by ASOS in 273 place over the country hourly. Quality was controlled following air pollution quality control manual (6). We used daily mean values.

References

1. Lee J, Lee JS, Park SH, et al. Cohort Profile: The National Health Insurance Service-National Sample Cohort (NHIS-NSC), South Korea. Int J Epidemiol 2017;46:e15.

2. Statistics Korea. Available at http://kosis.kr/eng/statisticsList/statisticsList_01List.jsp?vwcd=MT_ETITLE&parentId=B#SubCont Accessed July 24, 2016.

3. National Health Insurance Sharing Service. Available at http://nhiss.nhis.or.kr/ Accessed July 24, 2016.

4. Song SO, Jung CH, Song YD, et al. Background and data configuration process of a nationwide population-based study using the korean national health insurance system. Diabetes Metab J. 2014;38:395-403.

5. Korean Meteorological Administration. KMA Weather Data Service. Open MET Data Portal. Available at https://data.kma.go.kr/cmmn/main.do Accessed July 24, 2016.

6. Ministry of Environment. Public Open Data Service. Available at http://www.me.go.kr/home/web/index.do?menuId=10259 Accessed July 24, 2016.

S2 Table Pearson's correlation coefficients (r) between each of meteorological and air pollution variables for 3 years (1,095 days)

	Mean temperature	Highest temperature	Lowest temperature	Relative humidity	Pressure	Rainfall	Sunshine duration	SO_2	NO_2	O_3	CO	PM_{10}
Mean temperature	1.000	0.802*	0.936*	-0.456*	0.348*	0.293*	0.321*	0.055*	-0.131*	0.167*	-0.407*	-0.334*
Highest temperature	0.802*	1.000	0.543*	-0.183*	0.341*	-0.024*	0.265*	-0.187*	-0.434*	0.302*	-0.395*	-0.290*
Lowest temperature	0.936*	0.543*	1.000	-0.525*	0.297*	0.441*	0.311*	0.211*	0.080*	0.046*	-0.338*	-0.279*
Relative humidity	-0.456*	-0.183	-0.525*	1.000	-0.060*	-0.530*	-0.347*	-0.182*	-0.509*	0.424*	-0.101*	0.010
Pressure	0.348*	0.341*	0.297*	-0.060*	1.000	0.395*	0.183*	0.210*	0.251*	-0.217*	-0.087*	0.203*
Rainfall	0.293*	-0.024*	0.441*	-0.530*	0.395*	1.000	0.225*	0.227*	0.623*	-0.547*	0.009	0.112*
Sunshine duration	0.321*	0.265*	0.311*	-0.347*	0.183*	0.225*	1.000	0.165*	-0.044*	0.159*	-0.322*	-0.233*
SO_2	0.055*	-0.187*	0.211*	-0.182*	0.210*	0.227*	0.165*	1.000	0.492*	-0.397*	0.324*	0.482*
NO_2	-0.131*	-0.434*	0.080*	-0.509*	0.251*	0.623*	-0.044*	0.492*	1.000	-0.881*	0.580*	0.556*
O_3	0.167*	0.302*	0.046*	0.424*	-0.217*	-0.547*	0.159*	-0.397*	-0.881*	1.000	-0.667*	-0.660*
CO	-0.407*	-0.395*	-0.338*	-0.101*	-0.087*	0.009	-0.322*	0.324*	0.580*	-0.667*	1.000	0.626*
PM_{10}	-0.334*	-0.290*	-0.279*	0.010	0.203*	0.112*	-0.233*	0.482*	0.556*	-0.660*	0.626*	1.000

* Pearson's correlation coefficient analysis, Significance at $P < 0.05$
Pressure: Ambient atmospheric pressure

S3 Table Crude and adjusted odd ratios (95% confidence interval, CI) of the meteorological and pollution matter (mean of 1 year [365 days] before index date) for thyroid cancer

Characteristics	Odds ratio for thyroid cancer (95% CI)					
	Crude †	P-value	Model 1 †‡	P-value	Model 2 †§	P-value
Mean temperature for 1 year (365 days) (˚C)	1.07 (1.04-1.10)	<0.001*	1.06 (1.03-1.09)	<0.001*		
Highest temperature for 1 year (365 days) (˚C)	1.07 (1.04-1.10)	<0.001*	1.06 (1.03-1.09)	<0.001*		
Lowest temperature for 1 year (365 days) (˚C)	1.05 (1.02-1.07)	<0.001*	1.04 (1.01-1.07)	0.003*		
Temperature range for 1 year (365 days) (˚C)	1.01 (0.98-1.05)	0.492	1.02 (0.98-1.05)	0.427		
Relative humidity for 1 year (365 days) (%)	1.00 (0.99-1.01)	0.499	1.00 (0.99-1.01)	0.941	1.01 (1.00-1.02)	0.011*
Ambient atmospheric pressure for 1 year (365 days) (hPa)	1.02 (1.01-1.03)	<0.001*	1.02 (1.01-1.03)	<0.001*	1.02 (1.01-1.03)	<0.001*
Sunshine duration for 1 year (365 days) (hr)	1.24 (1.16-1.33)	<0.001*	1.29 (1.20-1.39)	<0.001*	1.22 (1.12-1.31)	<0.001*
Rainfall for 1 year (365 days) (mm)	1.01 (0.98-1.05)	0.468	1.02 (0.99-1.06)	0.179		
SO_2 for 1 year (365 days) (0.01 ppm)	0.73 (0.52-1.02)	0.063	0.70 (0.50-0.99)	0.042*		
NO_2 for 1 year (365 days) (0.01 ppm)	0.98 (0.94-1.03)	0.440	1.00 (0.96-1.05)	0.883	1.26 (1.18-1.35)	<0.001*
O_3 for 1 year (365 days) (0.01 ppm)	1.20 (1.11-1.31)	<0.001*	1.19 (1.10-1.30)	<0.001*		
CO for 1 year (365 days) (ppm)	0.45 (0.29-0.68)	<0.001*	0.44 (0.29-0.69)	<0.001*		
PM_{10} for 1 year (365 days) (10 μg/m^3)	0.80 (0.76-0.85)	<0.001*	0.80 (0.76-0.84)	<0.001*	0.69 (0.64-0.74)	<0.001*

Abbreviations: CCI, Charlson comorbidity index; DBP, diastolic blood pressure; SBP, systolic blood pressure

* Conditional logistic regression model, Significance at P < 0.05

† Stratified model for age, sex, income, and region of residence

‡ A model 1 was adjusted for total cholesterol, SBP, DBP, fasting blood glucose, obesity, smoking status, alcohol consumption, and CCI score.

§ A model 2 was adjusted for total cholesterol, SBP, DBP, fasting blood glucose, obesity, smoking status, alcohol consumption, CCI score, temperature range, relative humidity, ambient atmospheric pressure, sunshine duration, SO_2, NO_2, O_3, CO, and PM_{10} using forward selection method.

S4 Table Crude and adjusted odd ratios (95% confidence interval, CI) of the meteorological and pollution matter (mean of 5 years [1,825 days] before index date) for thyroid cancer

Characteristics	Odds ratio for thyroid cancer (95% CI)					
	Crude †	P-value	Model 1 †‡	P-value	Model 2 †§	P-value
Mean temperature for 5 years (1,825 days) (˚C)	1.05 (1.02-1.09)	0.001*	1.04 (1.01-1.08)	0.009*		
Highest temperature for 5 years (1,825 days) (˚C)	1.04 (1.01-1.07)	0.008*	1.03 (1.00-1.07)	0.045*		
Lowest temperature for 5 years (1,825 days) (˚C)	1.04 (1.02-1.07)	0.001*	1.04 (1.01-1.06)	0.010*		
Temperature range for 5 years (1,825 days) (˚C)	0.98 (0.94-1.01)	0.173	0.98 (0.94-1.02)	0.247		
Relative humidity for 5 years (1,825 days) (%)	1.01 (1.00-1.02)	0.151	1.00 (0.99-1.02)	0.484	1.02 (1.00-1.03)	0.016*
Ambient atmospheric pressure for 5 years (1,825 days) (hPa)	1.02 (1.01-1.03)	<0.001*	1.02 (1.01-1.03)	<0.001*	1.02 (1.01-1.03)	<0.001*
Sunshine duration for 5 years (1,825 days) (hr)	1.13 (1.02-1.26)	0.022*	1.15 (1.03-1.28)	0.016*	1.21 (1.06-1.37)	0.005*
Rainfall for 5 years (1,825 days) (mm)	1.07 (1.02-1.12)	0.011*	1.08 (1.02-1.13)	0.004*		
SO_2 for 5 years (1,825 days) (0.01 ppm)	0.68 (0.48-0.97)	0.032*	0.67 (0.46-0.96)	0.028*		
NO_2 for 5 years (1,825 days) (0.01 ppm)	0.97 (0.93-1.02)	0.278	1.00 (0.95-1.05)	0.930	1.44 (1.33-1.56)	<0.001*
O_3 for 5 years (1,825 days) (0.01 ppm)	1.17 (1.08-1.28)	<0.001*	1.15 (1.06-1.26)	0.001*		
CO for 5 years (1,825 days) (ppm)	0.31 (0.21-0.47)	<0.001*	0.31 (0.21-0.48)	<0.001*		
PM_{10} for 5 years (1,825 days) (10 μg/m^3)	0.81 (0.77-0.86)	<0.001*	0.82 (0.77-0.86)	<0.001*	0.60 (0.55-0.65)	<0.001*

Abbreviations: CCI, Charlson comorbidity index; DBP, diastolic blood pressure; SBP, systolic blood pressure

* Conditional logistic regression model, Significance at $P < 0.05$

† Stratified model for age, sex, income, and region of residence

‡ A model 1 was adjusted for total cholesterol, SBP, DBP, fasting blood glucose, obesity, smoking status, alcohol consumption, and CCI score.

§ A model 2 was adjusted for total cholesterol, SBP, DBP, fasting blood glucose, obesity, smoking status, alcohol consumption, CCI score, temperature range, relative humidity, ambient atmospheric pressure, sunshine duration, SO_2, NO_2, O_3, CO, and PM_{10} using forward selection method.

S5 Table Subgroup analyses of crude and adjusted odd ratios (95% confidence interval, CI) of the meteorological and pollution matter (mean of 1 year [365 days] before index date) for thyroid cancer according to age, sex, income, and region of residence

Characteristics	Odds ratio for thyroid cancer (95% CI)	
	Model 2 †‡	P-value
Age < 60 years old (n = 14,985)		
Temperature range for 1 year (365 days) (˚C)	1.13 (1.07-1.19)	<0.001*
Ambient atmospheric pressure for 1 year (365 days) (hPa)	1.03 (1.01-1.04)	<0.001*
NO_2 for 1 year (365 days) (0.01 ppm)	1.41 (1.26-1.58)	<0.001*
O_3 for 1 year (365 days) (0.01 ppm)	1.39 (1.11-1.73)	0.004*
PM_{10} for 1 year (365 days) (10 μg/m^3)	0.69 (0.63-0.75)	<0.001*
Age ≥ 60 years old (n = 8,175)		
Relative humidity for 1 year (365 days) (%)	1.02 (1.00-1.04)	0.037*
Sunshine duration for 1 year (365 days) (hr)	1.19 (1.04-1.36)	0.013*
NO_2 for 1 year (365 days) (0.01 ppm)	1.50 (1.28-1.75)	<0.001*
O_3 for 1 year (365 days) (0.01 ppm)	1.57 (1.17-2.12)	0.003*
PM_{10} for 1 year (365 days) (10 μg/m^3)	0.74 (0.66-0.84)	<0.001*
Males (n = 4,965)		
Relative humidity for 1 year (365 days) (%)	1.04 (1.02-1.07)	<0.001*
NO_2 for 1 year (365 days) (0.01 ppm)	1.65 (1.43-1.89)	<0.001*
PM_{10} for 1 year (365 days) (10 μg/m^3)	0.55 (0.48-0.64)	<0.001*
Females (n = 18,195)		
Ambient atmospheric pressure for 1 year (365 days) (hPa)	1.03 (1.02-1.04)	<0.001*
Sunshine duration for 1 year (365 days) (hr)	1.21 (1.11-1.31)	<0.001*
NO_2 for 1 year (365 days) (0.01 ppm)	1.17 (1.09-1.25)	<0.001*
PM_{10} for 1 year (365 days) (10 μg/m^3)	0.73 (0.68-0.79)	<0.001*
Low income (n = 9,260)		
Relative humidity for 1 year (365 days) (%)	1.02 (1.00-1.03)	0.038*
Ambient atmospheric pressure for 1 year (365 days) (hPa)	1.02 (1.01-1.04)	0.001*
Sunshine duration for 1 year (365 days) (hr)	1.22 (1.08-1.38)	0.001*
NO_2 for 1 year (365 days) (0.01 ppm)	1.16 (1.05-1.29)	0.004*
PM_{10} for 1 year (365 days) (10 μg/m^3)	0.71 (0.64-0.79)	<0.001*
High income (n = 13,900)		
Ambient atmospheric pressure for 1 year (365 days) (hPa)	1.02 (1.01-1.04)	<0.001*
Sunshine duration for 1 year (365 days) (hr)	1.23 (1.11-1.36)	<0.001*
NO_2 for 1 year (365 days) (0.01 ppm)	1.24 (1.14-1.36)	<0.001*
CO for 1 year (365 days) (ppm)	2.73 (1.12-6.66)	0.028*
PM_{10} for 1 year (365 days) (10 μg/m^3)	0.66 (0.60-0.73)	<0.001*
Urban (n = 11,080)		
Sunshine duration for 1 year (365 days) (hr)	1.16 (1.03-1.31)	0.012*
SO_2 for 1 year (365 days) (0.01 ppm)	2.05 (1.07-3.93)	0.031*
CO for 1 year (365 days) (ppm)	10.27 (4.54-23.23)	<0.001*
PM_{10} for 1 year (365 days) (10 μg/m^3)	0.60 (0.52-0.69)	<0.001*
Rural (n = 12,080)		

Relative humidity for 1 year (365 days) (%)	1.06 (1.04-1.08)	<0.001*
Sunshine duration for 1 year (365 days) (hr)	1.33 (1.16-1.53)	<0.001*
NO_2 for 1 year (365 days) (0.01 ppm)	1.59 (1.39-1.82)	<0.001*
CO for 1 year (365 days) (ppm)	0.10 (0.04-0.26)	<0.001*
PM_{10} for 1 year (365 days) (10 $\mu g/m^3$)	0.74 (0.65-0.85)	<0.001*

Abbreviations: CCI, Charlson comorbidity index; DBP, diastolic blood pressure; SBP, systolic blood pressure

* Conditional logistic regression model, Significance at $P < 0.05$

† Stratified model for age, sex, income, and region of residence

‡ A model 2 was adjusted for total cholesterol, SBP, DBP, fasting blood glucose, obesity, smoking status, alcohol consumption, CCI score, temperature range, relative humidity, ambient atmospheric pressure, sunshine duration, SO_2, NO_2, O_3, CO, and PM_{10} using forward selection method.

S6 Table Subgroup analyses of crude and adjusted odd ratios (95% confidence interval, CI) of the meteorological and pollution matter (mean of 5 years [1,825 days] before index date) for thyroid cancer according to age, sex, income, and region of residence

Characteristics	Odds ratio for thyroid cancer (95% CI)	
	Model 2 †‡	P-value
Age < 60 years old (n = 14,985)		
Temperature range for 5 years (1,825 days) (˚C)	1.07 (1.01-1.12)	0.012*
Ambient atmospheric pressure for 5 years (1,825 days) (hPa)	1.03 (1.02-1.04)	<0.001*
NO_2 for 5 years (1,825 days) (0.01 ppm)	1.40 (1.28-1.54)	<0.001*
PM_{10} for 5 years (1,825 days) (10 μg/m^3)	0.60 (0.54-0.67)	<0.001*
Age ≥ 60 years old (n = 8,175)		
Relative humidity for 5 years (1,825 days) (%)	1.04 (1.02-1.06)	0.001*
Sunshine duration for 5 years (1,825 days) (hr)	1.24 (1.00-1.55)	0.049*
NO_2 for 5 years (1,825 days) (0.01 ppm)	1.53 (1.35-1.75)	<0.001*
CO for 5 years (1,825 days) (ppm)	0.20 (0.07-0.58)	0.003*
PM_{10} for 5 years (1,825 days) (10 μg/m^3)	0.65 (0.56-0.75)	<0.001*
Males (n = 4,965)		
Relative humidity for 5 years (1,825 days) (%)	1.06 (1.03-1.08)	<0.001*
NO_2 for 5 years (1,825 days) (0.01 ppm)	1.92 (1.63-2.25)	<0.001*
PM_{10} for 5 years (1,825 days) (10 μg/m^3)	0.48 (0.40-0.57)	<0.001*
Females (n = 18,195)		
Ambient atmospheric pressure for 5 years (1,825 days) (hPa)	1.03 (1.02-1.04)	<0.001*
Sunshine duration for 5 years (1,825 days) (hr)	1.19 (1.03-1.36)	0.015*
NO_2 for 5 years (1,825 days) (0.01 ppm)	1.30 (1.20-1.42)	<0.001*
PM_{10} for 5 years (1,825 days) (10 μg/m^3)	0.65 (0.59-0.71)	<0.001*
Low income (n = 9,260)		
Ambient atmospheric pressure for 5 years (1,825 days) (hPa)	1.03 (1.02-1.04)	<0.001*
NO_2 for 5 years (1,825 days) (0.01 ppm)	1.23 (1.09-1.38)	0.001
PM_{10} for 5 years (1,825 days) (10 μg/m^3)	0.64 (0.57-0.73)	<0.001*
High income (n = 13,900)		
Ambient atmospheric pressure for 5 years (1,825 days) (hPa)	1.02 (1.01-1.04)	<0.001*
NO_2 for 5 years (1,825 days) (0.01 ppm)	1.45 (1.33-1.59)	<0.001*
PM_{10} for 5 years (1,825 days) (10 μg/m^3)	0.59 (0.54-0.66)	<0.001*
Urban (n = 11,080)		
Temperature range for 5 years (1,825 days) (˚C)	0.87 (0.78-0.96)	0.008*
Relative humidity for 5 years (1,825 days) (%)	0.97 (0.96-0.99)	<0.001*
NO_2 for 5 years (1,825 days) (0.01 ppm)	1.26 (1.06-1.50)	0.008*
O_3 for 5 years (1,825 days) (0.01 ppm)	1.90 (1.36-2.66)	<0.001*
CO for 5 years (1,825 days) (ppm)	25.72 (7.93-83.46)	<0.001*
PM_{10} for 5 years (1,825 days) (10 μg/m^3)	0.48 (0.41-0.55)	<0.001*
Rural (n = 12,080)		
Temperature range for 5 years (1,825 days) (˚C)	1.14 (1.05-1.24)	0.003*
Relative humidity for 5 years (1,825 days) (%)	1.07 (1.04-1.09)	<0.001*
NO_2 for 5 years (1,825 days) (0.01 ppm)	2.01 (1.69-2.38)	<0.001*

CO for 5 years (1,825 days) (ppm)	0.05 (0.02-0.13)	<0.001*
PM_{10} for 5 years (1,825 days) (10 μg/m^3)	0.59 (0.48-0.72)	<0.001*

Abbreviations: CCI, Charlson comorbidity index; DBP, diastolic blood pressure; SBP, systolic blood pressure

* Conditional logistic regression model, Significance at P < 0.05

† Stratified model for age, sex, income, and region of residence

‡ A model 2 was adjusted for total cholesterol, SBP, DBP, fasting blood glucose, obesity, smoking status, alcohol consumption, CCI score, temperature range, relative humidity, ambient atmospheric pressure, sunshine duration, SO_2, NO_2, O_3, CO, and PM_{10} using forward selection method.
